# New Activities of the Nuclear Pore Complexes

**DOI:** 10.3390/cells10082123

**Published:** 2021-08-18

**Authors:** Richard W. Wong

**Affiliations:** 1 WPI-Nano Life Science Institute, Kanazawa University, Kanazawa 920-1192, Japan; rwong@staff.kanazawa-u.ac.jp; 2 Graduate School of Frontier Science Initiative, Kanazawa University, Kanazawa 920-1192, Japan; 3 Cell-Bionomics Research Unit, Institute for Frontier Science Initiative, Kanazawa University, Kanazawa 920-1192, Japan

**Keywords:** nuclear pore complex, nanomedicine, liquid–liquid phase separation, biomacromolecule, HS-AFM, nucleoporin, nanoimaging

## Abstract

Nuclear pore complexes (NPCs) at the surface of nuclear membranes play a critical role in regulating the transport of both small molecules and macromolecules between the cell nucleus and cytoplasm via their multilayered spiderweb-like central channel. During mitosis, nuclear envelope breakdown leads to the rapid disintegration of NPCs, allowing some NPC proteins to play crucial roles in the kinetochore structure, spindle bipolarity, and centrosome homeostasis. The aberrant functioning of nucleoporins (Nups) and NPCs has been associated with autoimmune diseases, viral infections, neurological diseases, cardiomyopathies, and cancers, especially leukemia. This Special Issue highlights several new contributions to the understanding of NPC proteostasis.

The nuclear pore complex (NPC) [1,2,3] is a nanoscale gatekeeper with a central, selective, cobweb-like barrier composed mainly of nucleoporins (Nups), which comprise intrinsically disordered (non-structured) regions (IDRs) with phenylalanine–glycine (FG) motifs (FG-Nups) [4,5,6,7,8]. A fully assembled NPC in vertebrates contains multiple copies of approximately 30 different Nups and has an estimated molecular mass of 120 MDa [9]. Despite our knowledge of the NPC structure, the molecular mechanisms underlying its regulatory roles remain to be understood [1,3]. Using high-speed atomic force microscopy (HS-AFM), we showed that FG-Nups are in a liquid–liquid phase separated (LLPS) state [10,11] in which rapid and transient intermediates of FG filaments and sometimes co-exist with a central plug during conformational changes in cancer cells and organoids [12]. The real-time HS-AFM images also suggested that the NPC central channel has a moist cobweb-like structure [13]. Our laboratory also explored additional functions of NPCs beyond nuclear transport [14,15] and found that nucleoporins Rae1 [16,17], Tpr [18,19,20], Nup358/RanBP2 [21], Nup62 [22], Nup58 [23], and Nup88 [24] play critical roles in maintaining spindle bipolarity, centrosome and mid-body homeostasis during cell division [25,26]. For instance, we provided several lines of evidence that the phosphorylation of the cohesin subunit, SMC1, stimulates binding to mitotic Rae1 [27,28], a phenomenon recently confirmed by others to form part of a mechanism of antimitotic catastrophe in early tumorigenesis [29]. Indeed, Rae1 is highly overexpressed in colon cancer [30]. We and others also showed NPCs and nuclear transport proteins play critical roles in various cancers [31,32,33,34], especially leukemia [35,36]. A decade ago, we showed that the nuclear pore protein, Tpr, involved in autophagy [37], which affects ependymoma [38] and myogenic differentiation [39] in mammalian cells. Recently, two studies further proved that NPCs are targeted for vacuolar degradation through selective autophagy [40,41]. On the basis of our above contributions to the understanding of NPC structure and function, we were invited to organize a Special Issue in *Cells* entitled “Nuclear Pore Complex in Nanomedicine”.

In this Special Issue, Yang et al. explore the functions of Nup62-like (Nup62l) mRNA in the pharyngeal arches (PA) during zebrafish development [42]. They demonstrate that CRISPR/Cas9-mediated gene knockout of Nup62l leads to defective PA characterized by a thinned and shortened pharyngeal region and a significant loss of pharyngeal cartilage [42]. They also show that the aberrant activation of a series of apoptotic pathways in Nup62l-mutants is closely associated with the inactivation of Wnt/β-catenin signaling [42].

The contribution by Okazaki et al. shows that Nup62 depletion induces cell cycle arrest before meiosis without CDK1 activation in *Drosophila* cells [42]. The ectopic over-expression of CycB, but not constitutively active CDK1, results in partial rescue of the cell cycle. Protein complexes containing CycB, Emb, and Nup62 are also identified in premeiotic spermatocytes. CycB, which temporally enters the nucleus, associates with Emb, and the complex is transported back through the central channel to the cytoplasm, where it interacts with the Nup62 complex [43].

Emerging evidence suggests a crucial role for interactions between HIV-1 proteins and host nucleoporins [44,45] in the import of the pre-integration complex into the nucleus and export of viral RNAs into the cytoplasm during viral replication [46]. Remarkably, HIV destabilizes the host nuclear pore complex to enter into and exit through the nucleus. Several papers suggest a crucial role of interactions between HIV-1 proteins and host nucleoporins that underlie the import of the pre-integration complex into the nucleus and export of viral RNAs into the cytoplasm during viral replication [44,45,46]. Shukla and Chauhan highlight the recent progress and challenges in developing a more effective antiviral arsenal by exploring critical host–HIV interactions involving NPCs and nucleoporins [46].

The last contribution examines the pathways used by SARS-CoV-2 and other viruses to invade the host nucleocytoplasmic transport system. Sajidah et al. discuss the viral and host factors involved in the nuclear import and export of viral components [47]. The novel human betacoronavirus SARS-CoV-2 has triggered an unprecedented pandemic in the 21st century, and several studies have revealed interactions between SARS-CoV-2 viral proteins and host nucleoporins [48,49]. As nucleocytoplasmic shuttling is crucial for the replication of many viruses, Sajidah et al. also review several drugs that target the host nuclear transport machinery and discuss their feasibility as antiviral treatments [47]. How viral components enter or hijack the NPC undoubtedly requires further exploration.

Unlike most studies of NPCs, which use yeast or mammalian cells as model systems, the above contributions to this short Special Issue investigate NPC protein Nup62 in zebrafish and *Drosophila* systems. In addition, several interesting NPC-related studies have also recently been published in *Cells*. For example, extracellular vesicles (EVs) derived from cancer cells contain proteins and nucleic acids responsible for pro-tumorigenic and pro-metastatic effects. What happens to EV cargo molecules once inside the target cells? Does nuclear delivery of the endocytosed EV components impair intercellular crosstalk? Are there any other pathways besides nuclear pores implicated in the nuclear translocation of EV-derived cargo? [50]. Interaction between endocytosed EVs and NPCs may provide another target for therapeutic intervention using nanomedicine in the near future.

Finally, the direct visualization of NPCs and DNA–histone interactions is the key to understanding epigenetic regulation and gene expression [51]. NPCs and nuclear envelope proteins are involved in modulating heterochromatin formation and functions in fission yeast [52] and regulating chromatin state [53]. The condensation and compartmentalization of membraneless biomacromolecules such as NPCs and their FG-Nups are dependent on phase separation. Analytical tools using microfluidic devices, nanoimaging systems with high spatiotemporal resolution such as HS-AFM [54], and proteomics, together with artificial intelligence methods (e.g., Alphafold2) [55,56] that combine them, are expected to play an important role in studying the NPC-LLPS-mediated control of chromatin structure [57]. An understanding of this crucial area of NPC function will be required for a definitive proof of the gene gating hypothesis proposed by Gunter Blobel [58].
